# Supplementary material for: Effective Synthesis of Carbon Hybrid Materials Containing Oligothiophene Dyes
Source: Materials (Basel). 2019 Oct 15;12(20):3354. doi: 10.3390/ma12203354 (PMC6829365; doi:10.3390/ma12203354)
Supplement: Supplementary file 1 [file materials-12-03354-s001.pdf]

materials

Supporting Materials

Effective Synthesis of Carbon Hybrid Materials Containing Oligothiophene Dyes

Piotr Kamedulski[1*], Piotr A. Gauden[1], Jerzy P. Lukaszewicz[1,2] and Anna Ilnicka[1]

[1] Faculty of Chemistry, Nicolaus Copernicus University, Gagarina 7, 87-100 Torun, Poland
[2] Centre for Modern Interdisciplinary Technologies, Wilenska 4, 87-100 Torun, Poland
* Correspondence: pkamedulski@umk.pl

Section: Results in experiments

The low pressure nitrogen adsorption isotherms of pristine carbon and hybrid materials are shown in Figure S1. The morphology of the surface of the obtained carbon materials was analysed using a scanning electron microscopy analysis (SEM). Representative images of the obtained carbon materials are collected in Figures S2 and S3. SEM/EDX images presented in Figure S4 confirm the presence of sulphur in the carbon matrixes after impregnation with oligothiophene dye 3T.

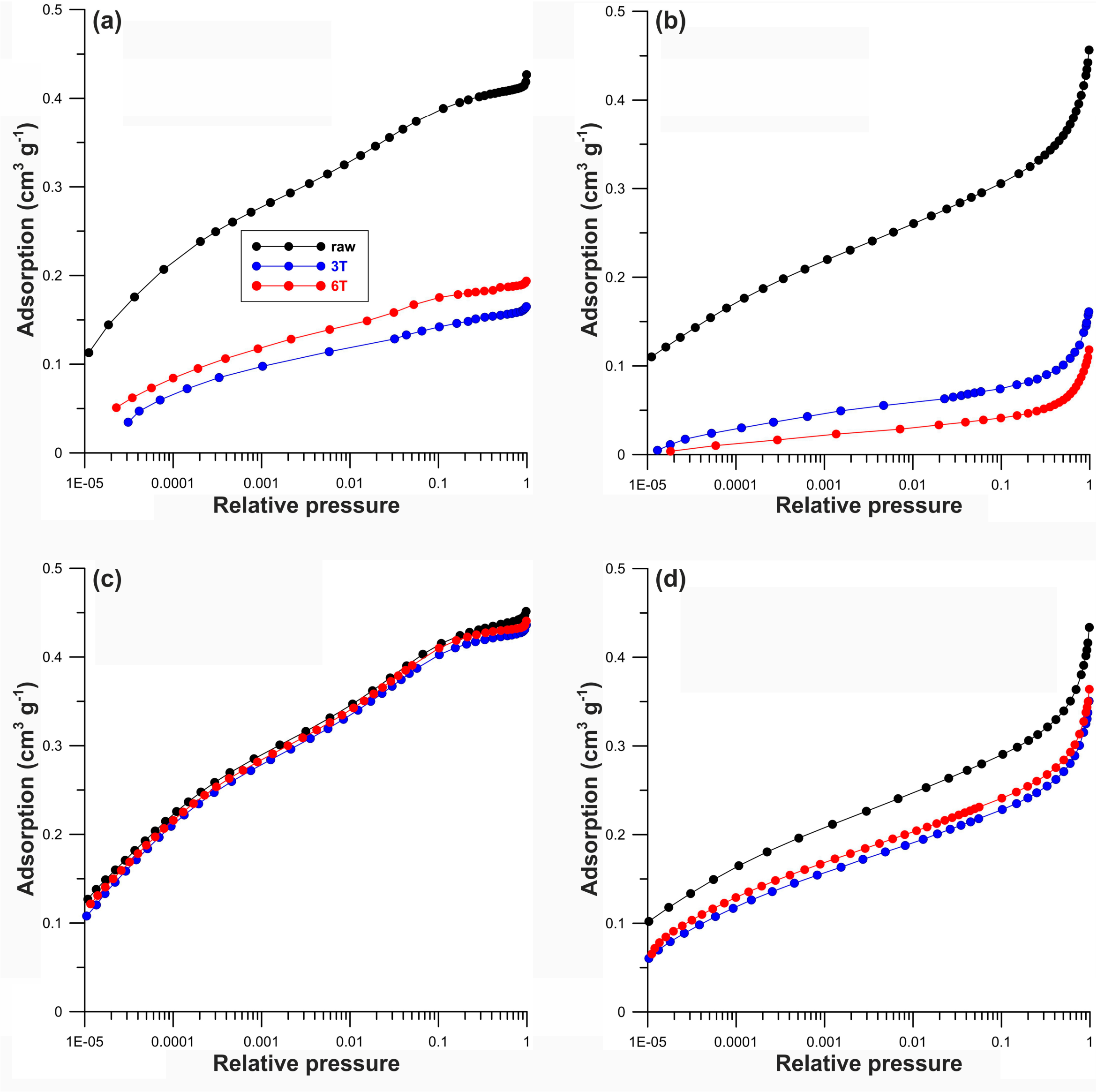

Figure S1. Nitrogen adsorption isotherms of pristine carbon and hybrid materials for: (a) Norit_1, (b) Norit_2, (c) Norit_1_ox, and (d) Norit_2_ox.

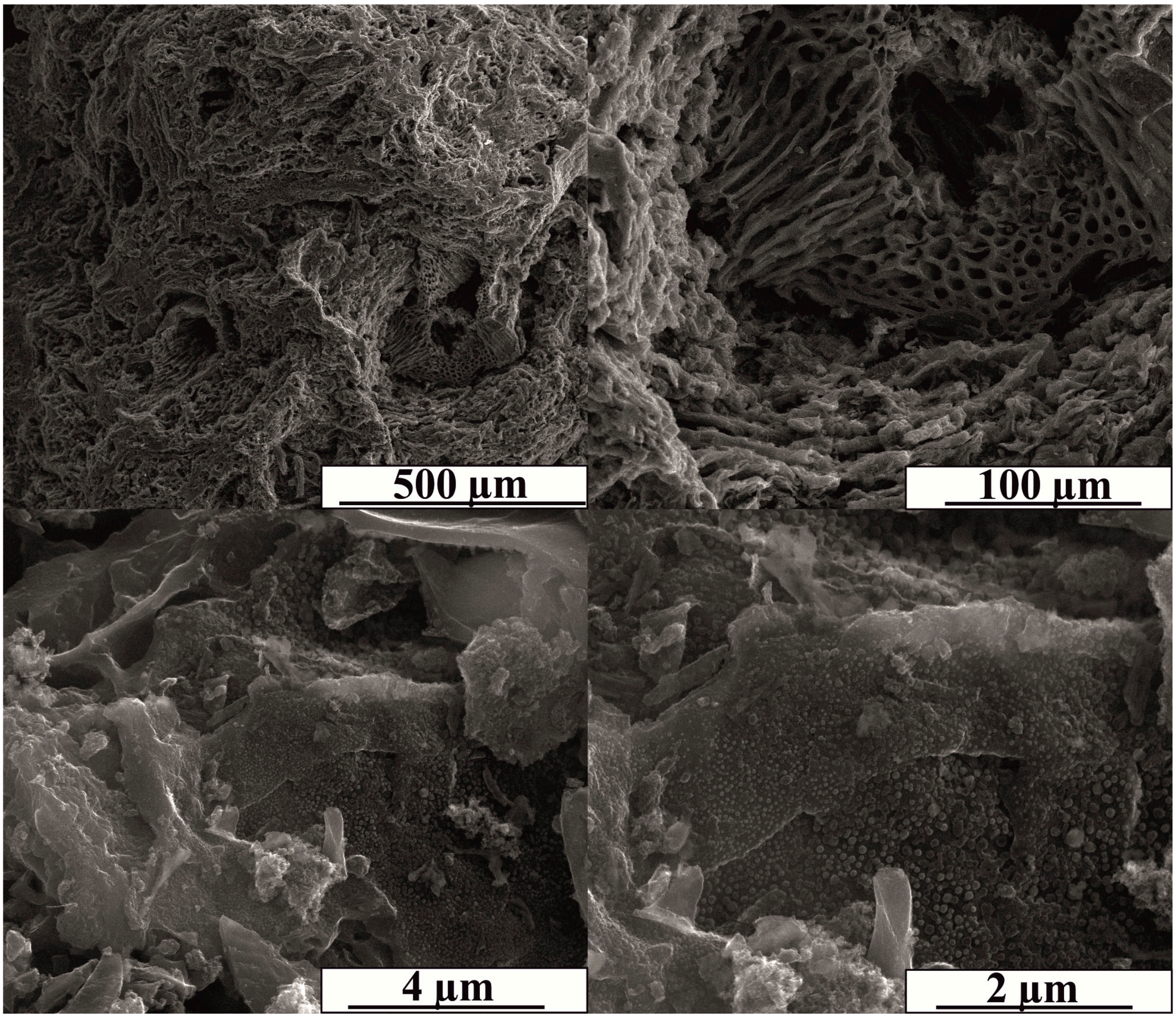

Figure S2. SEM images of Norit_2_3T sample in different magnifications.

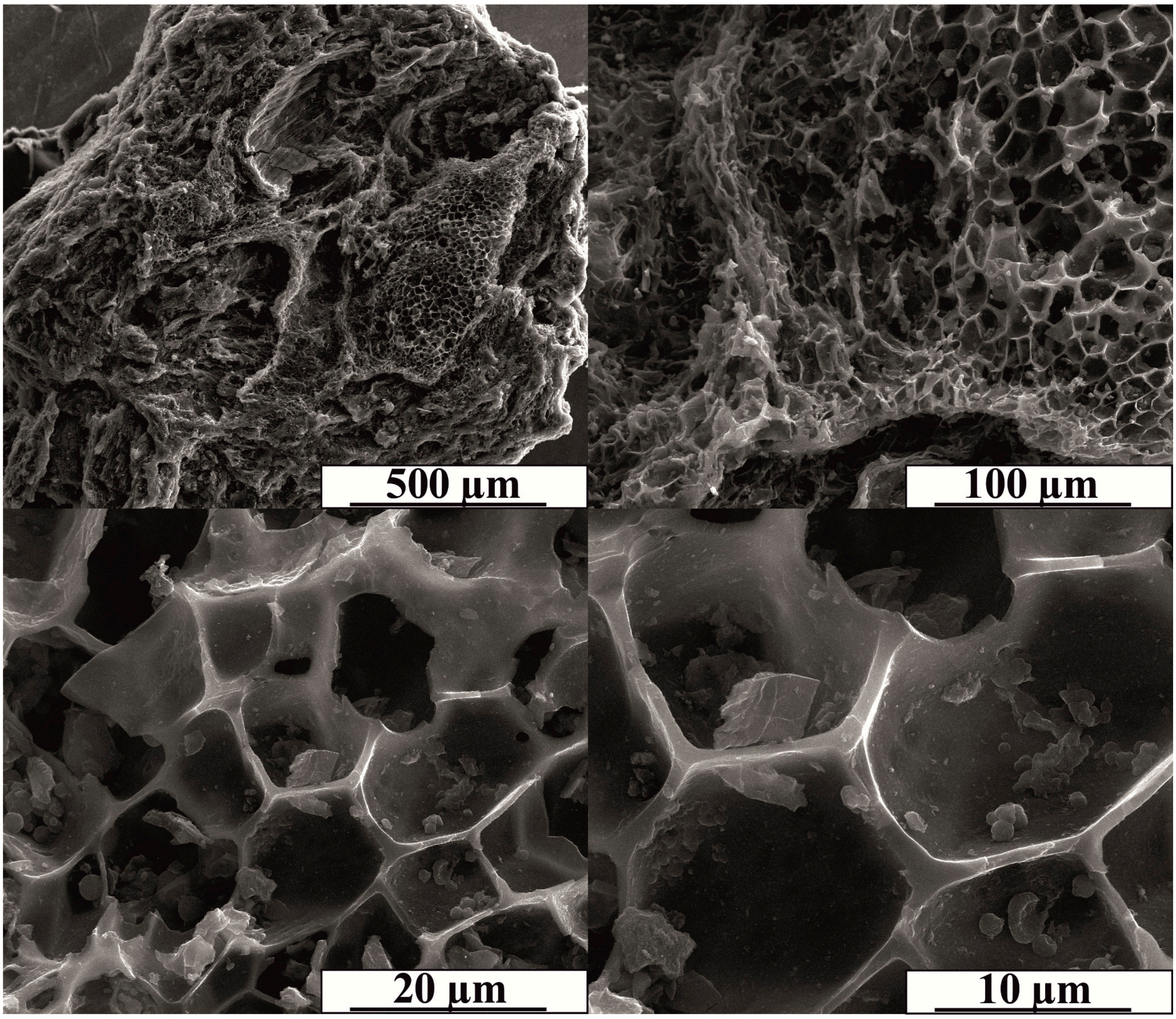

Figure S3. SEM images of Norit_2_ox_3T sample in different magnifications.

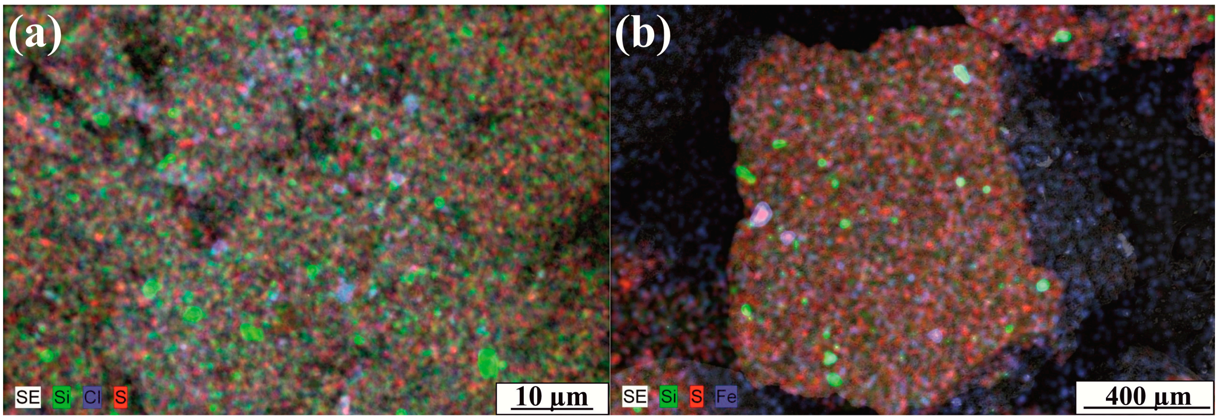

Figure S4. SEM/EDX mapping images of: (a) Norit_1_3T and (b) Norit_1_ox_3T samples.

© 2019 by the authors. Submitted for possible open access publication under the terms and conditions of the Creative Commons Attribution (CC BY) license (http://creativecommons.org/licenses/by/4.0/).
